# Motor Variability during Sustained Contractions Increases with Cognitive Demand in Older Adults

**DOI:** 10.3389/fnagi.2014.00097

**Published:** 2014-05-22

**Authors:** Marnie L. Vanden Noven, Hugo M. Pereira, Tejin Yoon, Alyssa A. Stevens, Kristy A. Nielson, Sandra K. Hunter

**Affiliations:** ^1^Exercise Science Program, Department of Physical Therapy, Marquette University, Milwaukee, WI, USA; ^2^Department of Psychology, Marquette University, Milwaukee, WI, USA

**Keywords:** arousal, ankle dorsiflexor muscles, muscle fatigue, age, motor variability, steadiness, aging, stress

## Abstract

To expose cortical involvement in age-related changes in motor performance, we compared steadiness (force fluctuations) and fatigability of submaximal isometric contractions with the ankle dorsiflexor muscles in older and young adults and with varying levels of cognitive demand imposed. Sixteen young (20.4 ± 2.1 year: 8 men, 9 women) and 17 older adults (68.8 ± 4.4 years: 9 men, 8 women) attended three sessions and performed a 40 s isometric contraction at 5% maximal voluntary contraction (MVC) force followed by an isometric contraction at 30% MVC until task failure. The cognitive demand required during the submaximal contractions in each session differed as follows: (1) *high-cognitive demand* session where difficult mental math was imposed (counting backward by 13 from a 4-digit number); (2) *low-cognitive demand* session which involved simple mental math (counting backward by 1); and (3) *control session* with no mental math. Anxiety was elevated during the high-cognitive demand session compared with other sessions for both age groups but more so for the older adults than young adults (*p * < 0.05). Older adults had larger force fluctuations than young adults during: (1) the 5% MVC task as cognitive demand increased (*p * = 0.007), and (2) the fatiguing contraction for all sessions (*p * = 0.002). Time to task failure did not differ between sessions or age groups (*p * > 0.05), but the variability between sessions (standard deviation of three sessions) was greater for older adults than young (2.02 ± 1.05 vs. 1.25 ± 0.51 min, *p * < 0.05). Thus, variability in lower limb motor performance for low- and moderate-force isometric tasks increased with age and was exacerbated when cognitive demand was imposed, and may be related to modulation of synergist and antagonist muscles and an altered neural strategy with age originating from central sources. These data have significant implications for cognitively demanding low-force motor tasks that are relevant to functional and ergonomic in an aging workforce.

## Introduction

Aging results in marked declines in both motor performance and cognitive function. For example, older adults are weaker and less steady (i.e., they exhibit greater fluctuations in force around a target force) than young adults (Enoka et al., 2003; Hunter et al., 2008). Decreased steadiness with age is greatest during low-intensity isometric contractions in lower and upper extremity muscles (Enoka et al., 2003; Tracy, 2007; Marmon et al., 2011) probably due to age-related changes in inputs to the motoneurone pool (Barry et al., 2007). Cognitive impairment can also be marked but subtle and often subclinical in early stages of cognitive dysfunction (Chen et al., 2001; Morris et al., 2001; Aine et al., 2011); it is often observed as degradation in short-term memory and executive function resulting in a decreased ability to perform daily tasks that are dependent upon memory-related abilities (Artero et al., 2001; Morris et al., 2001; Farias et al., 2012), including planning and decision-making in lower limb activities such as gait (Yogev-Seligmann et al., 2008). Age-related declines in motor and cognitive function are usually studied separately but are often performed simultaneously in daily tasks. The current study assessed motor function with a focus on steadiness and muscle fatigability in young and older adults while they were presented with low and high levels of cognitive demand.

In young adults, cognitive performance declines and force fluctuations increase when a cognitive task is imposed (reaction time) during sustained isometric tasks with hand muscles (Lorist et al., 2002; Zijdewind et al., 2006); however, force fluctuations were affected more during the isometric fatiguing contractions than during a 5% maximal voluntary contraction (MVC) non-fatiguing contraction (Lorist et al., 2002). We also previously found that steadiness of the elbow flexor muscles declined (increased force fluctuations) and time to failure of a sustained 20% MVC submaximal task was reduced (increased fatigability) in young adults when simultaneously performing a demanding cognitive task (‘high-cognitive demand’) that increased anxiety (counting backwards by 13). Accordingly, heart rate and blood pressure, which are indices of increased sympathetic activity with arousal (Kajantie and Phillips, 2006), were elevated during the “high-cognitive demand” session compared with control (Yoon et al., 2009; Keller-Ross et al., 2014a). Individuals who were weaker (primarily the women) showed the largest decrement in time to task failure when the stressful cognitive task was imposed during the fatiguing contraction (Yoon et al., 2009; Keller-Ross et al., 2014a). Older adults are typically weaker than young adults, and older women weaker than older men for upper and lower limb muscles (Galganski et al., 1993; Laidlaw et al., 2000; Tracy and Enoka, 2002), possibly increasing susceptibility to increased fatigability when a cognitive task is imposed. However, it is not known whether fatigability with increased cognitive demand is exacerbated with advanced age. Furthermore, the effects of increased cognitive demand on lower limb fatigability and steadiness in young or older adults are not known.

Initial evidence would suggest that age-related decrements in motor function of the upper limb (e.g., reduced steadiness) become larger with greater cortical involvement of non-motor centers (Voelcker-Rehage et al., 2006; Voelcker-Rehage and Alberts, 2007; Fraser et al., 2010) and may increase between- and within-participant variability particularly in older adults (Enoka et al., 2003; Sosnoff and Newell, 2006). Older adults display greater between- and within-participant variability than young in activation of supraspinal centers during maximal contractions with the upper limb (Hunter et al., 2008) indicating that variability within cortical motor areas is larger with advanced age. Increased cognitive involvement, particularly with tasks that tax attentional resources and that require the use of short-term memory or executive function, may contribute further to age-related variability in motor performance for older adults during functional tasks (Voelcker-Rehage et al., 2006; Yogev-Seligmann et al., 2008; Sommervoll et al., 2011).

Increased cognitive involvement however, can increase anxiety and stress levels (e.g., Noteboom et al., 2001), which may further decrease steadiness of upper limb muscles in older adults compared with young. Force fluctuations, for example, increased for older adults more than young when exposed to a noxious stressor (unpredictable electrical stimulation to the hand) prior to performing the pinch grip task (Christou et al., 2004). The increased force fluctuations therefore, could be due to age-related changes in monoaminergic drive modulating inputs to the motoneurone pool (Barry et al., 2007). A novel aspect of this current study is that we varied the level of cognitive demand administered simultaneously during motor task of the lower limbs to determine its influence on lower limb fatigability and steadiness and any accompanying changes in anxiety and stress.

The purpose of this study was to compare both the amplitude of force fluctuations (steadiness) and time to task failure (fatigability) for low-to-moderate-force isometric contractions in the presence and absence of varying levels of cognitive demand in young and older adults. Participants were exposed to two different levels of cognitive load, low- and high-cognitive demand while performing a motor task with the ankle dorsiflexor muscles, which are muscles that play a functional role controlling the position of the foot during walking and while maintaining balance. We *hypothesized* that older adults would show greater reductions in time to task failure and greater force fluctuations than young as cognitive demand increased. We also compared variability of fatigability between and within young and older adults with increased cognitive demand. We *hypothesized* that as cognitive demand increased, older adults would exhibit both greater between- and within-participant variability in fatigability. To understand the perceived and physiological arousal responses with the varying levels of cognitive demand in young and older adults, during each session we quantified perceived levels of exertion, stress and anxiety as well as heart rate and blood pressure.

## Materials and Methods

Sixteen young adults (8 men, 8 women; 18–24 years) and 17 older adults (9 men, 8 women; 62–79 years) participated in the study (see Table 1 for physical characteristics). All participants were healthy with no known neurological or cardiovascular diseases, had controlled blood pressure and were naïve to the protocol. Six of the older adults were on medication to control blood pressure. Both young and older adults had low-to-moderate levels of anxiety (trait) according to the State-Trait Anxiety Inventory (STAI) (Spielberger et al., 1970) and reported no history of current mental or psychological pathology, including anxiety or depressive disorders. Participants were right-leg dominant (0.81 ± 0.40 vs. 0.80 ± 0.54 for young and older adults, respectively, with a ratio of 1 indicating complete dominance of the right leg) (Oldfield, 1971). The physical activity level for each participant was assessed with a questionnaire that estimated the relative kilocalorie expenditure of energy per week (Kriska and Bennett, 1992). Prior to participation, each participant provided informed consent, and the protocol was approved by the Institutional Review Board at Marquette University.

**Table 1:** **Demographic and physical characteristics and age group means (±SD) for control, low-cognitive demand (low-CD), and high-cognitive demand (high-CD) sessions**.

Variable	Session	Young	Older
Number of participants		16	17
Age (years)		20.4 ± 2.1	68.8 ± 4.4^a^
Height (cm)		168.9 ± 23.6	167.5 ± 11.3
Women age (years)		19.3 ± 1.5	68.4 ± 3.6^a^
Men age (years)		21.5 ± 2.0	69.2 ± 4.9^a^
Weight (kg)		74.4 ± 27.9	77.8 ± 15.2
Physical activity (METS hours per week)		59.5 ± 38.3	22.0 ± 21.8^a^
Baseline trait STAI scores		36.4 ± 7	34.8 ± 7.3
Baseline state STAI scores		26.0 ± 6.7	26.2 ± 5.3
MVC torque (Nm)	Control	22.4 ± 6.9	19.1 ± 6.6
	Low-CD	22.2 ± 6.6	18.3 ± 5.8
	High-CD	22.8 ± 7.7	18.7 ± 6.3
	Total	22.5 ± 7.0	18.7 ± 6.1^a^
MVC torque recovery (% of initial)	Control	96.3 ± 7.4	97.9 ± 6.8
	Low-CD	95.4 ± 3.7	93.2 ± 8.5
	High-CD	94.8 ± 3.9	94.7 ± 5.5
	Total	96.2 ± 7.4	97.6 ± 6.5
Time to task failure (min)	Control	6.1 ± 2.2	8.0 ± 3.2
	Low-CD	6.1 ± 2.0	7.6 ± 3.1
	High-CD	7.1 ± 2.5	8.7 ± 3.8
	Total	6.4 ± 2.2	8.1 ± 3.4
30% MVC CV of torque (%)	Control	4.5 ± 1.2	6.0 ± 3.0
	Low-CD	4.5 ± 0.8	7.2 ± 4.1
	High-CD	4.6 ± 2.7	6.8 ± 2.3
	Total	4.5 ± 1.0	6.7 ± 3.2^a^
TA EMG^a^ (% MVC)	Control	23.5 ± 5.6	29.1 ± 6.0
	Low-CD	23.5 ± 6.2	27.2 ± 4.7
	High-CD	24.1 ± 4.8	28.7 ± 8.4
	Total	23.7 ± 4.3	28.3 ± 5.9^a^
TA EMG bursting activity (bursts per min)	Control	10.3 ± 9.7	5.7 ± 5.7
	Low-CD	11.5 ± 10.6	9.0 ± 9.2
	High-CD	9.4 ± 9.6	7.7 ± 7.6
	Total	10.4 ± 9.8	7.4 ± 7.6
Soleus EMG (% MVC)	Control	20.3 ± 9.6	28.4 ± 14.2
	Low-CD	19.2 ± 5.3	28.8 ± 12.2
	High-CD	17.4 ± 6.1	30.0 ± 13.9
	Total	18.9 ± 7.2	29.1 ± 13.2^a^
Gastrocnemius EMG (% MVC)	Control	26.7 ± 15.8	32.9 ± 13.8
	Low-CD	26.3 ± 9.9	30.6 ± 11.8
	High-CD	21.1 ± 6.1	29.9 ± 13.8
	Total	24.2 ± 11.3	31.1 ± 12.9^a^
Rectus femoris EMG (% MVC)	Control	5.4 ± 3.8	17.3 ± 22.0
	Low-CD	5.3 ± 4.2	18.5 ± 22.9
	High-CD	5.0 ± 3.0	15.8 ± 20.8
	Total	5.8 ± 3.8	17.2 ± 21.5^a^

*^a^Variables reaching statistical significance for main effect of age (*p* < 0.05). STAI, State-Trait Anxiety Index; MVC, maximal voluntary contraction; TA, tibialis anterior; EMG, electromyography; METS hours per week = metabolic equivalents hours per week*.

**Figure 1:**
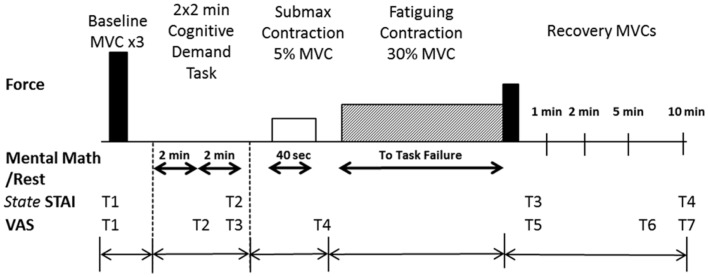
**Experimental protocol**. The top panel illustrates in order the tasks performed by each participant with the ankle dorsiflexor muscles. Maximal voluntary contractions (MVC) (solid bars) were performed at the beginning of the experimental session and during recovery (immediately after the fatiguing contraction and at 1, 2, 5, and 10 min of recovery). The fatiguing contraction (30% MVC), symbolized by the hatched rectangle, was performed until task failure by each participant. The bottom panels indicate when the State-Trait Anxiety Inventory (STAI; four times) was performed and the Visual Analog Scale (VAS) for anxiety and stress were recorded (at seven time points, T1–T7). The schematic is not to scale for time or force.

Each participant reported to the laboratory on four occasions to perform a protocol that involved a fatiguing contraction with the left ankle dorsiflexor muscles: once for a familiarization session and three experimental sessions (*control*, *low-cognitive demand*, and *high-cognitive demand*), with each experimental session being at least 5 days apart. During the low-cognitive demand and high-cognitive demand sessions, each participant performed either a simple mental math task (low-cognitive demand session) or difficult mental math task (high-cognitive demand session) at rest, and also while performing isometric contractions at 5% maximum voluntary contraction (MVC) force (40 s duration) and a 30% MVC for as long as possible until task failure (Figure 1). During the control session, each participant performed the motor tasks without performing any mental math. Session order was counterbalanced among participants within each age group.

### Mechanical recording of force

Each participant was seated upright in an adjustable chair (Biodex Medical Systems, NY, USA) with the hips and knees at 90° of flexion. The setup is similar to that described elsewhere (Griffith et al., 2010). In brief, the left foot rested on a footplate in a custom made dynamometer to measure forces of the lower leg, with the ankle in a neutral position (0° dorsiflexion). The foot was secured to the footplate via a strap placed over the anterior aspect of the ankle and another strap placed 1–2 cm proximal to the metatarsophalangeal joint. Isometric force of the dorsiflexor muscles was recorded using a force transducer (Transducer Techniques, Temecula, CA, USA) and recorded online at 500 Hz using a Power 1401 analog-to-digital (A/D) converter and Spike2 software [Cambridge Electronics Design (CED), Cambridge, UK]. Force displayed on a 19-inch monitor was located at eye level 1.5 m in front of the participant. Each participant was asked to trace a horizontal cursor placed in the middle of the screen with the force signal as it appeared on the screen from the right side of the monitor.

### Electrical recordings

Whole muscle electromyographic (EMG) signals of the tibialis anterior, medial head of the gastrocnemius, soleus, and rectus femoris were recorded using bipolar surface electrodes (sintered pellet Ag-AgCl, 8 mm diameter, 20 mm between electrodes) taped to the skin over the belly of each muscle. Reference electrodes were placed on the patella. The recording electrodes on each muscle were placed in line with the muscle fibers and in accordance with locations recommended by the European Recommendations for Surface Electromyography (Hermens et al., 2000). The EMG signals were amplified (1,000 ×) and band-pass filtered (13–1,000 Hz) with Coulbourn bioamplifiers (Coulbourn Instruments, Allentown, PA, USA) prior to being recorded directly to a computer using the Power 1401 and Spike2 software (CED). The EMG signals were digitized at 2,000 samples/s and analyzed offline using Spike2 software (CED).

### Cardiovascular measurements

Heart rate and blood pressure were monitored during submaximal and fatiguing contractions and periods of rest or mental math with an automated beat-by-beat blood pressure monitor (Finapress 2300, Datex-Ohmeda, Louisville, CO, USA). The blood pressure cuff was placed around the middle finger of the left hand, and the arm was placed on a platform to maintain the hand at heart level. Blood pressure was sampled at 500 samples/s and collected online to PC using Spike2 software (CED).

### Cognitive assessment of anxiety and stress

Cognitive levels of anxiety and stress were assessed throughout the protocol using a visual analog scale (VAS) (Yoon et al., 2009) and the *state* portion of the STAI questionnaire (Spielberger et al., 1970). Each VAS (one for anxiety and another for stress) had a 10-cm line anchored at the far left by “none” and at the far right by “as bad as it could be.” The right anchor corresponded to the most stressful or most anxious moment in the life of the participant. Anxiety was defined as the participant’s negative feelings regarding the immediate future, whereas stress represented the physical changes (e.g., increase in heart rate and perspiration) occurring during the test perceived by the participant that were above and beyond the expectation for their level of exertion (Christou et al., 2004). VAS for anxiety and stress were recorded at seven time points (T1–T7) during the protocol: one baseline assessment before intended arousal (T1); during the rest period after each 2 × 2-min bout of mental math (low-cognitive demand or high-cognitive demand session) or quiet rest (control session) (T2, T3); immediately after the 5% MVC submaximal contraction (T4); immediately after the fatiguing contraction/MVC (T5); and 5 and 10 min after the fatiguing contraction (T6, T7) (Figure 1).

The *state* portion of the STAI-questionnaire consisted of 20 statements that required a response on a four-point Likert-type scale. Baseline *trait* and *state* assessments were conducted during the familiarization session. There was no significant difference between young and older adults in baseline *trait* STAI scores (*p* = 0.54) or baseline *state* STAI scores taken during the familiarization session (*p * = 0.66) (Table 1). *State* STAI assessments were also conducted at four different time points during the experimental protocol: baseline assessment before arousal; after 2 × 2-min bouts of quiet sitting (control session) or mental math (low-cognitive demand and high-cognitive demand sessions); immediately after the fatiguing contraction/MVC; and 10 min after completion of the fatiguing contraction (Figure 1).

### Cognitive demand conditions

*Difficult mental math* is an established psychosocial technique used to induce cognitive demand and increase levels of anxiety (Kajantie and Phillips, 2006) and was used for the *high-cognitive demand task* (Noteboom et al., 2001). Each participant performed serial subtraction from a four-digit number by 13 with one response required every 3 s (Noteboom et al., 2001). If the participant made an error in serial subtraction or was unable to provide the correct answer within 3 s, they were asked to restart the mental math from the first number in the series. After three errors, the investigator asked the participant to begin with a new four-digit number. The *simple mental math task*, performed during the *low-cognitive demand session*, was designed to increase cognitive demand above control without elevating arousal. Participants serially counted backward by 1’s from 50 to 0 at a slow, even pace. If the participant made an error in counting, they were asked to restart counting from 50. During the *control session*, participants were instructed to rest quietly during the 2 × 2-min bouts, 5% MVC submaximal contraction (40 s) and 30% MVC fatiguing contraction. During the low-cognitive demand and high-cognitive demand sessions, participants performed the mental math task while at rest (2 × 2-min bouts), and then continuously during the 5% MVC submaximal contraction and 30% MVC fatiguing contraction until task failure.

### Experimental protocol

The protocol for each experimental session (control, low-cognitive demand, and high-cognitive demand sessions) involved procedures in the following order: (1) MVCs of the ankle dorsiflexor, ankle plantarflexor, and knee extensor muscles; (2) assessment of cognitive and physiological arousal before and after 2 × 2-min bout of either quiet sitting (control session), simple mental math (low-cognitive demand session), or difficult mental math (high-cognitive demand session); (3) performance of one submaximal isometric contraction at 5% MVC force sustained for 40 s with assessment of cognitive and physiological arousal immediately following the contraction; (4) a submaximal fatiguing isometric contraction at 30% MVC force sustained until task failure; and (5) recovery MVCs immediately following the fatiguing contraction, and at 1, 2, 5, and 10 min recovery with assessment of anxiety and stress levels (Figure 1).

Participants performed two MVCs of the knee extensor and plantar flexor muscles each at the beginning of each experimental session in order to obtain peak EMG for the gastrocnemius, soleus, and rectus femoris muscles. Participants rested for 60 s between each trial. For both muscle groups, MVCs were performed with the participant seated in the same position as used for testing the ankle dorsiflexors muscles (described above). The aim was to obtain peak EMG values for each muscle group; forces were not recorded during knee extension and plantar flexion. Each participant was asked to push as hard as possible against an immovable restraint for 3–4 s to activate either the knee extensor or ankle plantar flexor muscles. For the knee extensor muscles, manual resistance was applied to the distal leg (just above the lateral malleolus) so that the lower leg was restrained at 90° of flexion while the participant performed maximal knee extension. For the ankle plantar flexor muscles, the foot of each participant was placed on the footplate, and vertical movement was minimized during each MVC by a block that eliminated movement of the footplate. The MVC trial with the greatest amount of EMG activity was used to normalize the EMG recordings of the rectus femoris, medial head of the gastrocnemius, and soleus muscles during the submaximal contractions.

Participants performed three to four MVC trials with the ankle dorsiflexors while their foot was attached to the footplate. Each participant was asked to dorsiflex as hard as possible for 3–4 s. Participants were given visual feedback on a display monitor and strong verbal encouragement to achieve and maintain maximal force. Participants rested for 60 s between each trial. If the peak force achieved for two of the first three trials was not within 5% of each other, additional trials were performed until this criterion was met. The greatest MVC force achieved with the ankle dorsiflexor muscles was used as the reference to calculate the target level for both the submaximal contractions at 5% MVC and the fatiguing contraction at 30% MVC. The MVC with the greatest amount of EMG activity was used to normalize the EMG recordings during the fatiguing contractions of the tibialis anterior muscle. MVCs of the ankle dorsiflexor muscles were also performed during recovery (Figure 1).

A fatiguing contraction was performed with the ankle dorsiflexor muscles at 30% MVC during each experimental session. Each participant was asked to trace a horizontal cursor with the force signal as it appeared from the right side of the monitor in order to match the vertical target force as displayed on the screen. Participants were encouraged to sustain the force for as long as possible. The fatiguing contraction was terminated when the force declined by 10% of the target force. To minimize the influence of transient fluctuations in motor output on the criteria for task failure, the task was terminated only after force fell below the predetermined threshold for 2.5 s of a 5 s interval. Participants were not informed of their time to task failure.

Rating of perceived exertion (RPE) was assessed using the modified Borg 10-point scale (Borg, 1982). Each participant was instructed to focus their assessment of exertion on the ankle muscles performing the fatiguing task. The scale was anchored so that 0 represented the resting state and 10 corresponded to the strongest contraction that the ankle muscles could perform. The RPE was recorded at the beginning of the fatiguing contraction and every minute thereafter until task failure. In order to obtain RPE while the participant was performing mental math during the low-cognitive demand and high-cognitive demand sessions, the participant was interrupted and asked to report their RPE. After reporting their RPE, participants resumed the mental math task of serial counting from 50 to 0 during the low-cognitive demand session or serial subtraction using a new four-digit number during the high-cognitive demand session.

### Data analysis

All data collected during the experiments were recorded online using a Power 1401 A/D converter and analyzed using Spike2 (CED). The MVC torque was quantified as the average value over a 0.5-s interval that was centered about the peak. The torque for the MVCs, submaximal and fatiguing contractions was calculated as the product of force and the distance between the ankle joint and the point at which the ankle was attached to the force transducer. The maximal EMG for each muscle was determined as the root mean square (RMS) value over a 0.5-s interval about the same peak interval of the MVC torque measurement. The maximal EMG value of the involved muscles was then used to normalize the RMS EMG values recorded during the 5% MVC task and the fatiguing contraction at 30% MVC. The RMS value of the 5% MVC task was averaged for each muscle over the middle 30 s of the 40 s contraction for the tibialis anterior, medial gastrocnemius, soleus, and rectus femoris. During the fatiguing contraction, the RMS EMG signal for each muscle was quantified at the following time intervals: the first 30 s; 15 s on both sides of 25, 50, and 75% of time to task failure; and the last 30 s of the task duration. The EMG activity of each muscle was normalized to the RMS EMG value obtained during the MVC for each respective muscle.

To quantify the bursts of EMG activity of the tibialis anterior during the fatiguing contraction at 30% MVC, the EMG signal was first rectified, smoothed (averages of 1 s duration, 500 data points), and then differentiated over 0.25 s averages. The differentiated signal represents the rate of change and was used to identify rapid changes in the rectified and smoothed EMG signal. The threshold for establishing if a burst of EMG had occurred was determined by first finding the minimum standard deviation (SD) of the differentiated EMG during the fatiguing contraction using a 30 s moving window; the threshold was then defined as the mean + 3 SD of the minimum differentiated signal. The minimal burst duration was 0.1 s. The EMG bursting activity (bursts/min) was quantified for five continuous intervals of 20% of the time to task failure.

The amplitude of the force fluctuations was quantified as the coefficient of variation (CV) (CV = SD/mean × 100) for the 5% MVC task and 30% MVC fatiguing contraction. The fluctuations in torque during the 5% MVC task were quantified over the middle 30 s of the 40 s contraction. For the fatiguing contraction at 30% MVC, the fluctuations in torque were quantified for five continuous intervals of 20% of the time to task failure (between 0 and 100%).

Mean arterial pressure (MAP) and heart rate were evaluated only on participants not currently taking blood pressure medications with normal blood pressure. MAP and heart rate recorded during the fatiguing contraction at 30% MVC were analyzed by comparing ~15 s averages at 25% intervals throughout the fatiguing contraction; during the 5% MVC submaximal contraction they were quantified over the middle 30 s of the 40 s contraction. For each interval, the blood pressure signal was analyzed for the mean peaks [systolic blood pressure (SBP)], mean troughs [diastolic blood pressure (DBP)], and number of pulses per second (multiplied by 60 to determine heart rate). MAP was calculated for each epoch with the following equation: MAP* * = DBP + 1/3 (SBP−DBP). Rate-pressure product (RPP) was calculated as the product of heart rate and MAP for the equivalent time periods as stated above.

### Statistical analysis

Data were reported as means ± SD within the text, and displayed as means ± SE in the figures. Analyses of variances (ANOVA) models were used to compare the various dependent variables. Specifically, separate ANOVAs with repeated measures on session (control, low-cognitive demand, and high-cognitive demand), and with age (young and old) and sex (men and women) as fixed factors, were used to compare MVC torque and STAI (state) and VAS for anxiety and stress at baseline, MAP, heart rate, CV of torque, and EMG activity during the 5% MVC task, error rates (only low- and high-cognitive demand sessions included for error rate analysis) during the fatiguing contraction and the time to task failure of the fatiguing contraction. This ANOVA model was also used to compare the SD of the time to failure from the three sessions, and the SD of the mean CV of torque during the fatiguing contraction obtained from the three sessions. ANOVAs with repeated measures on session and time, and with age and sex as fixed factors, were used to compare VAS for stress and anxiety, STAI (state) and MVC torques throughout the sessions, i.e., before and after the fatiguing contraction (see Figure 1 for time points). ANOVAs with repeated measures on session and time (five time points for each: see Data Analysis), and with age and sex as fixed factors, were used to compare the following variables during the fatiguing contraction: CV of torque, RMS EMG, EMG bursting activity, heart rate, MAP, RPP, and RPE. *Post hoc* analyses (Tukey) were used to test for differences among pairs when appropriate. Univariate ANOVAs were used to compare young and old men and women for the following variables: physical characteristics (age, height, and weight), physical activity level, handedness, and STAI (trait). The strength of an association is reported as the Pearson product–moment correlation coefficient (*r*). The statistical significance adopted was 5% (*p* < 0.05) and all analysis were performed in IBM Statistical Package for Social Sciences (SPSS) version 19.

## Results

Young and older adults were of similar height and weight (*p* > 0.05) but the older adults were less active than the young (*p* < 0.05). See Table 1 for subject characteristics.

### MVC torque

At baseline, young adults were stronger than the older adults (22.5 ± 7 vs. 18.7 ± 6.1 Nm, respectively: age effect, *p * = 0.005) and men were stronger than women (sex effect, *p* = 0.0001) on all 3 days of testing, with no interaction between sex and age (age × sex, *p* = 0.69) (Table 1). The relative reduction (%) in MVC torque after the fatiguing contraction was similar across sessions (session effect, *p* = 0.98), and similar for young and older adults (session × age, *p* = 0.26). During recovery, MVC torque increased to near baseline levels within 10 min of completing the fatiguing contraction similarly for young and older adults across all sessions (session × age, *p* = 0.57) (Table 1). Furthermore, at the end of the recovery period, the MVC (% of baseline) was similar for young and older men and women (*p* > 0.05).

### Anxiety and stress levels

#### State STAI scores

Baseline *state* STAI scores taken at the beginning of each experimental session were similar for young and older men and women (age effect, *p* = 0.88; sex effect, *p* = 0.57) (Table 1). *State* STAI scores taken after exposure to the 2 × 2 min of difficult mental math were higher during the high-cognitive demand session compared with the control and low-cognitive demand sessions (control, 25.1 ± 6.6; low-cognitive demand, 30.4 ± 10.0; high-cognitive demand, 42.8 ± 13.1; and session effect, *p* = 0.0001) and immediately after the fatiguing contraction (control, 36.2 ± 9.2; low-cognitive demand, 38 ± 9; high-cognitive demand, 50.4 ± 13.8; and session effect, *p* = 0.0001). There was no difference between young and older adults across all three session after completing the fatiguing contraction (age effect, *p* = 0.72). Older adults however, demonstrated higher *state* STAI scores than young adults after the fatiguing contraction when exposed to the high-cognitive demand (session × time × age, *p* = 0.02).

#### VAS for stress and anxiety

**Figure 2:**
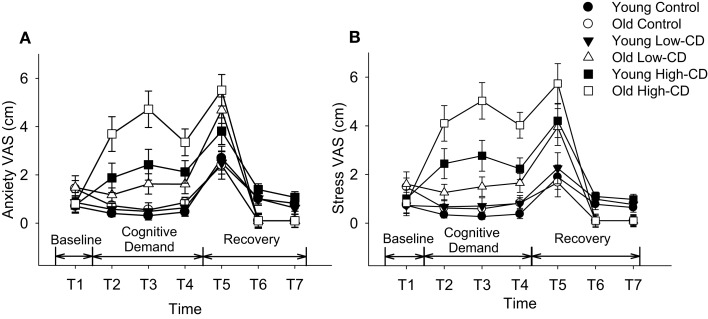
**Visual Analog Scale (VAS) scores for anxiety (A) and stress (B)**. Mean (±SE) VAS scores for young adults (closed symbols) and older adults (open symbols) are shown for anxiety **(A)** and stress **(B)** throughout the experimental protocol during the control session (circles), low-cognitive demand session (Low-CD, triangles), and high-cognitive demand session (High-CD, squares). Time intervals were as follows: baseline (T1), after the first bout of 2 min of quiet rest/mental math (T2), after the second bout of 2 min of quiet rest/mental math (T3), after the 5% submaximal contraction (T4), during recovery immediately after task failure (T5), and then at 5 min (T6) and 10 min of recovery (T7).

Visual analog scale scores for stress and anxiety were similar at baseline for young and older adults, and men and women (*p* > 0.05, Figure 2). *Anxiety VAS* was significantly higher during the high-cognitive demand session compared to the control and low-cognitive demand sessions (session × time, *p* = 0.0001), and increased more for older adults than young over time (time × age, *p* = 0.01) (Figure 2A). There were no other interactions. *Stress VAS* scores were significantly higher for older adults than young during the high-cognitive demand session compared with the control and low-cognitive demand sessions (session × age, *p* = 0.02, Figure 2B) and for older adults than young over time (time × age, *p* = 0.001). There were no main effects of sex and no other interactions.

### Low-intensity sustained contraction (5% MVC)

#### Fluctuations in torque

**Figure 3:**
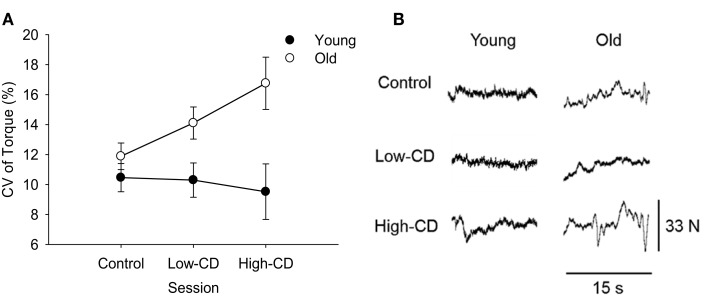
**Coefficient of Variation (CV) of torque during the 5% MVC task for young (closed symbols) and older (open symbols) adults during the control, low-cognitive demand (Low-CD), and high-cognitive demand (High-CD) sessions (A), and representative force tracings of a young and older adult (B)**. Shown are the mean (±SE) in **(A)**. Older adults had significantly higher CV of torque than young adults (age effect, *p * = 0.007).

Older adults had greater fluctuations in torque (CV of torque) compared with young across all three sessions (age effect, *p* = 0.007) (Figure 3) and women had higher fluctuations in torque than men (sex effect, *p * = 0.001). On average, the older adults had a linear increase in fluctuations in torque as cognitive demand increased while young adults showed no change (linear interaction for session × age, *p* = 0.04, Figure 3A). Women demonstrated higher force fluctuations than men (*p* = 0.001), but there were no other interactions (*p* > 0.05).

#### MAP and heart rate

Cardiovascular measures were analyzed for only those older participants who were *not* currently taking blood pressure medications at the time of the experiment (young, *n* = 16; older, *n* = 11). During the 5% MVC task, MAP was higher during the high-cognitive demand session (105.1 ± 18.8 mmHg) than the low-cognitive demand session (87.2 ± 28.3 mmHg) and the control session (88.8 ± 23 mmHg; session effect, *p * = 0.001). There was no influence of age or sex on MAP (*p* > 0.05). Similarly, heart rate was greater during the high-cognitive demand session (79.6 ± 15 beats/min) compared with the low-cognitive demand (71.6 ± 28 beats/min) and control sessions (68.2 ± 19.4 beats/min, session effect, *p * = 0.04) regardless of age of sex (*p* > 0.05, i.e., no interactions). Consequently, the *RPP* was higher during the high-cognitive demand session than the control and low-cognitive demand sessions (session effect, *p* = 0.0001), but there was no difference between young and older adults (age effect, *p* = 0.52) or men and women (sex effect, *p * = 0.70).

#### EMG activity

During the 5% MVC task, older adults had higher soleus RMS EMG (% MVC) (antagonist muscle) than young adults during the high-cognitive demand session (session × age, *p* = 0.04), but there were no other interactions, or main effects of age, sex or session for soleus or any other muscles (tibialis anterior, gastrocnemius, and rectus femoris).

### Fatiguing contraction (30% MVC)

#### Time to task failure

**Figure 4:**
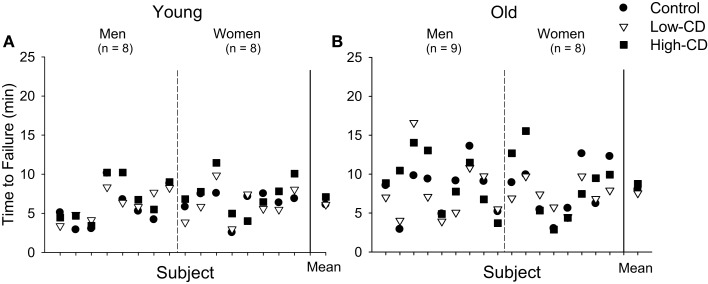
**Time to task failure during the fatiguing contraction for individual young (A) and older adults (B) during the control (circle), low-cognitive demand (Low-CD, triangle), and high-cognitive demand (High-CD, square) sessions**. Time to task failure is shown for each young **(A)** and older **(B)** man and woman for each session (separated by dashed line). The aggregate mean (±SE) is shown for each session after the solid vertical line. The range and variability of time to task failure among the older adults was greater than for the young adults for each session. Older adults were more variable than young between the three sessions due to addition of a cognitive task (age effect, *p* = 0.03), but variability did not increase with task difficulty (session effect, *p* = 0.38).

There was no difference in time to task failure across sessions or with age (*p* > 0.05) (Table 1; Figure 4). There was no difference in time to task failure between men and women (sex effect, *p* = 0.96) and no interactions for age, sex, and session (*p* > 0.05). Variability between the three sessions in the time to task failure (comparison of SD generated from the three sessions for each participant) however, was greater for older adults than young adults (2.02 ± 1.05 vs. 1.25 ± 0.51 min, respectively, *p* = 0.02), but similar for men and women (sex effect, *p* = 0.53; sex × age, *p* = 0.57) (Figure 4). Furthermore, we compared the SD for time to task failure between sessions (SD for control and low-cognitive demand session vs. SD for control and high-cognitive demand session) to determine if variability increased with difficulty of the mental math. While the age effect remained (age effect, *p* = 0.03), there were no effects of session (session effect, *p* = 0.38) or sex (sex effect, *p* = 0.69), and no interactions. Thus, although the older adults were more variable than young between the three sessions due to addition of a cognitive task, the variability between the older adults did not increase with to the difficulty of the cognitive task.

#### Fluctuations in torque

**Figure 5:**
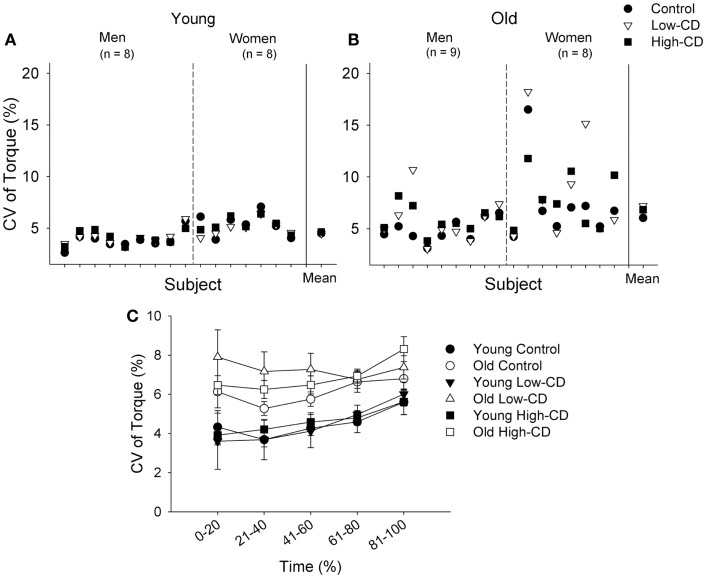
**Coefficient of variation of torque during the fatiguing contraction (30% MVC) for individual young (A) and older adults (B), and across time for young and older adults (C) during the control session (circle), low-cognitive demand session (Low-CD, triangle), and high-cognitive demand session (High-CD, square)**. CV of torque is shown for each young **(A)** and older **(B)** man and woman for each session. The aggregate mean (± SE) is shown for each session after the solid vertical line. The older adults and women exhibited greater variability in steadiness across sessions than the young and men respectively (*p * < 0.05). In **(C)**, the CV of torque (mean ± SE) is shown for the control (circle), low-CD demand (triangle), and high-CD (square) sessions for young (closed symbols) and older (open symbols) adults during five continuous intervals of 20% during the fatiguing contraction between 0 and 100% of the time to failure. Thus, the *x* axis intervals for **(C)** indicate the interval of data points averaged in each 20% interval. Older adults had significantly higher CV of torque than young adults (age effect, *p * = 0.002).

Fluctuations in torque (CV) increased over time during the sustained contraction (time, *p * < 0.001) similarly across sessions for both age groups (session × time, *p * = 0.56, Figure 5C). Older adults however, had larger fluctuations in torque than young adults (age effect, *p * = 0.002) and this difference was similar across sessions (session × age, *p* = 0.11). Women also had larger fluctuations in torque than men (sex effect, *p* = 0.005) and this sex difference was similar across sessions (session × sex, *p* = 0.69). There were no other interactions (*p* > 0.05). Because older and young adults can demonstrate more variability in motor performance than young adults, we evaluated variability of the fluctuations in torque (CV of torque) during the 30% MVC task (comparison of SD generated from the three sessions for each participant) between young and older adults. Variability was greater for older adults than young adults (*p* = 0.01), and greater for women than men (sex effect, *p* = 0.02), but there were no interactions (sex × age, *p* = 0.94) (Figures 5A,B).

### EMG activity of agonist muscles

The amplitude of the RMS EMG (% MVC) for the tibialis anterior (ankle dorsiflexors), increased during the fatiguing contraction (time effect, *p * < 0.001) and similarly across sessions (session × time, *p * = 0.38). Furthermore, tibialis anterior EMG activity was greater for the older adults than young during all three sessions (age effect, *p * = 0.002) (Table 1), but there was no difference between men and women (sex effect, *p * = 0.80), and no interactions.

Electromyographic bursting activity (bursts per minute) increased over time during the fatiguing contraction (30% MVC) for all three sessions (time effect, *p * = 0.002); however, there was no difference across the sessions (session effect, *p * = 0.50). Neither age nor sex influenced the bursting activity during the fatiguing contraction (*p* > 0.05, Table 1).

### EMG activity of antagonist and accessory muscles

The RMS EMG amplitude (% MVC) for the soleus, gastrocnemius, and rectus femoris increased over time during the fatiguing contractions (time effect, *p* < 0.001 for all muscles) but was similar across sessions (session effect, *p* > 0.05 for all muscles). Older adults however, had higher RMS EMG for the soleus (age effect, *p * = 0.04), gastrocnemius (*p * = 0.01), and rectus femoris (*p* = 0.03) than young adults (see Table 1). There was no effect of sex for gastrocnemius and soleus (sex effect, *p* > 0.05) and there were no interactions (*p* > 0.05). For the rectus femoris, however, women had higher RMS EMG amplitudes than men (sex effect, *p * = 0.04), but there were no interactions (*p* > 0.05). There was a correlation between rectus femoris RMS EMG and CV of torque for the high-cognitive demand session only, indicating that participants who had greater torque fluctuations during the high-cognitive demand session also had greater rectus femoris EMG (*r*_33_ = 0.31, *p* = 0.04).

### MAP and heart rate

**Figure 6:**
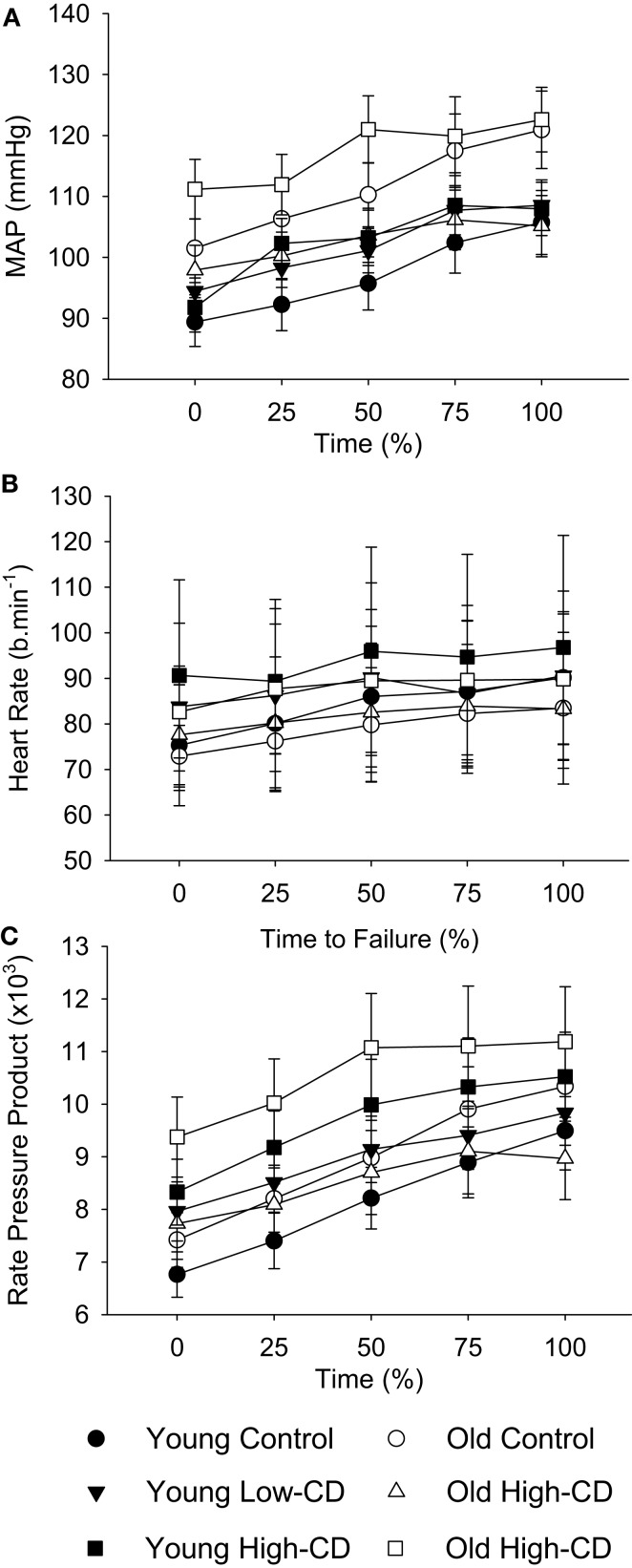
**Mean arterial pressure, heart rate, and rate-pressure product during the fatiguing contraction for young and older adults across sessions**. The values are mean ± SE at 25% increments of time to task failure for young (closed symbols) and older (open symbols) adults during the control (circles), low-cognitive demand (Low-CD, triangles), and high-cognitive demand (High-CD, squares) sessions for mean arterial pressure **(A)**, heart rate **(B)**, and rate-pressure product **(C)** during the fatiguing contraction. Averages of 15-s intervals were used for the MAP and heart rate. Rate-pressure product was the product of heart rate and MAP for the equivalent time periods in**(A,B)**.

Cardiovascular measures were included only for older participants who were not currently taking blood pressure medications at the time of the experiment (young, *n* = 16; older, *n* = 11). MAP, heart rate, and RPP increased over time (time, *p* < 0.05) (Figure 6). MAP, heart rate, and RPP were higher during the high-cognitive demand session than the control or low-cognitive demand sessions (session effect, *p* < 0.05). MAP was higher for older adults during the fatiguing contraction in the high-cognitive demand session over time than other sessions (session × time × age, *p* = 0.02). Heart rate and RPP, however, were similar for older adults over time across sessions (session × time × age, *p* > 0.05), with no main effects of age or sex (*p* > 0.05).

#### Rating of perceived exertion (RPE)

Perceived exertion increased during the fatiguing contraction (time effect, *p* = 0.0001) similarly across sessions (session effect, *p* = 0.59). RPE was similar for young and older adults (age effect, *p* = 0.82) and men and women (sex effect, *p* = 0.62), with no interactions (*p* > 0.05). Mean RPE across all three sessions was 4.2 ± 1.5 and 4.2 ± 1.8 at the beginning of the fatiguing contraction for young and older adults respectively and increased to 8.7 ± 2.3 vs. 9.1 ± 1.4 by the end of the fatiguing contraction.

#### Error rate

The mental math error rate during the fatiguing contraction (errors/min) was significantly higher during the high-cognitive demand session (2.9 ± 1.3 errors/min) compared with the low-cognitive demand session (0.4 ± 0.4 errors/min, session effect, *p* < 0.001). There was no main effect of age or sex (*p* > 0.05). There was also no correlation between error rate and CV of torque during the fatiguing contraction for the low-cognitive demand (*r*_33_ = −0.17, *p* = 0.35) or high-cognitive demand (*r*_33_ = −0.26, *p* = 0.14) sessions; nor were there significant associations between error rate during the fatiguing contraction and the time to task failure for the low-cognitive demand (*r*_33_ = −0.12, *p* = 0.52) or high-cognitive demand (*r*_33_ = −0.06, *p* = 0.74) sessions.

## Discussion

This study imposed several levels of cognitive demand during sustained low- and moderate-force isometric contractions with the ankle dorsiflexor muscles to determine the influence of increased cortical involvement on motor function and fatigability in young and older adults. The novel findings of this study were that as cognitive demand increased, steadiness decreased (i.e., CV of torque increased) during the very low-force contraction (5% MVC) for older adults but did not change for the young adults. While fatigability (time to failure) of a moderate intensity contraction (30% MVC) was not essentially different with imposed cognitive demand for young or older adults, variability in the time to failure and in the torque fluctuations across sessions was greater for the older adults than the young adults. These results provide evidence that increased cortical involvement of motor and non-motor cortical areas can disrupt motor performance of low-to-moderate intensity isometric contractions of the lower limb more so in older adults than young adults.

### Steadiness was reduced with age in the lower limb

Torque fluctuations (CV) were greater (steadiness reduced) during the low-intensity contraction (5% MVC) than at the start of the 30% MVC task (prior to fatigue), and also greater for older adults than young across all sessions. Larger torque fluctuations with advanced age have also been observed under control conditions across various muscle groups and particularly at the lower intensity contractions for both young and older men and women (Enoka et al., 2003; Tracy et al., 2005, 2007). Typically, the CV (%) will decrease as contraction intensity increases (Enoka et al., 2003; Taylor et al., 2003; Moritz et al., 2005; Tracy, 2007) and as we observed. Because we showed increased torque fluctuations during ankle dorsiflexion for the older adults during both 5% MVC and 30% MVC tasks compared with young, the age-related mechanism for reduced steadiness under control conditions influences both the low- and moderate-force tasks in the ankle dorsiflexor muscles. CV of torque across a range of low-to-high forces appear to be primarily modulated by low-frequency oscillations (<2–3 Hz) in neural drive found in motor unit action potentials trains (Negro et al., 2009; Dideriksen et al., 2012) with some contribution from increased motor unit variability at very low forces (Dideriksen et al., 2012; Jesunathadas et al., 2012). This low-frequency oscillating neural drive likely reflects an integration of both descending and afferent inputs onto the motoneurone pool (Negro et al., 2009; Dideriksen et al., 2012; Farina et al., 2012). With advanced age, the motoneurone pool undergoes remodeling that results in decreased motor units numbers and altered relations between discharge rates and recruitment thresholds (Barry et al., 2007); the age difference in torque fluctuations, therefore, appears to be due to age-related changes in the inputs to the motoneurone pool (Barry et al., 2007) with possibly some influence of greater motor unit discharge rate variability in older adults (Laidlaw et al., 2000; Kornatz et al., 2005; Tracy et al., 2005; Barry et al., 2007). Age-related changes in visual-motor processing may also contribute to altered motoneuronal inputs causing increased torque fluctuations during static contractions with age (Henningsen et al., 1997; Seidler-Dobrin and Stelmach, 1998; Tracy et al., 2007; Fox et al., 2013).

### Cardiovascular responses and anxiety were elevated with high-cognitive demand

While mental math was used to manipulate different levels of cognitive demand in this study, it can also increase anxiety and stress (Kajantie and Phillips, 2006) as it did for the young but more so in the older adults during the high-cognitive demand task (see Figure 2). Accordingly, MAP and heart rate were elevated when the difficult mental math was performed during both the 5% MVC and 30% MVC tasks, although similarly for the young and older adults. Older adults have reduced maximal heart rates compared with young, explaining the similar age-related increase in heart rate despite older adults reporting they felt more anxious and stressed. Because both MAP and heart rate were elevated, rate-pressure product was elevated for both young and older adults indicating increased cardiac work and myocardial oxygen consumption (Gobel et al., 1978; Wasmund et al., 2002) during the 5% MVC task and 30% MVC fatiguing contraction when cognitive demand was high. Chronicity of high blood pressure has been associated with an increased risk of stroke, cognitive decline, and dementia, especially in older adults with untreated high blood pressure (Tzourio et al., 1999; Tzourio, 2007). In the short term, increased stress and anxiety can increase monoaminergic drive; neuromodulatory inputs alter excitability of the motoneurone pool (Heckman et al., 2009) and potentially alter motor neuron output especially at low forces.

### Steadiness decreased with cognitive demand in older adults

A novel finding was that the age difference in CV of torque grew linearly (i.e., steadiness decreased) with the increased levels of cognitive demand during a low-intensity contraction (5% MVC) of the lower limb. Although steadiness during the 30% MVC task was similar across sessions, older adults had greater variability in the CV of torque between sessions than the young. Increased torque fluctuations during the 5% MVC task between the low- and high-cognitive demand sessions indicate that the decline in steadiness was not solely due to the added distraction or challenge of talking because the low-cognitive demand task controlled for those factors. Because motor unit discharge rate variability can contribute to the force fluctuations at very low forces (Tracy et al., 2005; Jesunathadas et al., 2012), increased variability of the motor unit pool in the older adults may have been altered when high-cognitive demand was imposed.

Our findings indicate that increased antagonist muscle activation may also have contributed to the larger force fluctuations in the older adults when cognitive demand was high during the 5% MVC task. The older adults had greater soleus muscle activation during the high-cognitive demand session relative to the other sessions and compared with the young adults: this suggests less inhibition of the antagonist muscle from descending cortical sources. Increased antagonist muscle activity is a strategy adopted by older adults to stiffen joints and reduce movement variability with age (Hortobagyi and DeVita, 2006). For the higher force task (30% MVC) the torque fluctuations were larger with age, but there was no increase in the CV of torque with cognitive demand for young or older adults. Agonist (tibialis anterior), antagonist (gastrocnemius and soleus), and synergist (rectus femoris) activations were greater in the older adults than the young across all sessions, possibly contributing to the larger torque fluctuations with age. Because both the agonist (tibialis anterior) and antagonist activation were greater in the older adults compared with the young, activation differences had minimal effect on the greater CV of torque in the older adults.

There are numerous age-related changes along the neuroaxis that can alter inputs to the motoneurone pool and perhaps make it more susceptible to altering motor output when cognitive demand is imposed. Cortical size (Raz et al., 2007) and processing are diminished with age, along with reduced corticospinal fibers numbers (Eisen et al., 1996), and changes in spinal reflex pathways (Kido et al., 2004), which result in decreased cortical inhibition of both cognitive and motor processes with age (Peinemann et al., 2001; Sale and Semmler, 2005; Hunter et al., 2008). Age-related changes along the neuroaxis can also result in increased activation of antagonist muscles (Macaluso et al., 2002; Hortobagyi and DeVita, 2006) and as discussed may have been responsible for the greater activation of antagonist muscles with age in the current study.

Several theories of motor control assert that the division of attentional resources during a dual-task paradigm has limits and these limitations increase with advanced aging due to diminished cortical processing (Woollacott and Shumway-Cook, 2002). Consequently, some studies show older adults have less ability to simultaneously perform a cognitive task and motor task as well as they can be performed individually (Voelcker-Rehage and Alberts, 2007; Fraser et al., 2010; Johnson and Shinohara, 2012). Changes in performance for older adults in dual-tasks appear to be especially sensitive to cognitive tasks that require executive function (Yogev-Seligmann et al., 2008). Executive function (which included working memory, which was varied in this study), anxiety, and stress are modulated in prefrontal cortical regions and the anterior cingulate cortex (Miller, 2000; Owen et al., 2005; Banich et al., 2009; Schweizer et al., 2013). Prefrontal connections to motor areas (Takahara et al., 2012) along with input to neural connections between these and other cortical centers associated with cognition, anxiety, and motor function could directly alter motor output, as was observed in this study.

Capacity theories of attention that assume attentional resource limitations on the ability to perform multiple tasks simultaneously (Kahneman, 1973; McDowd, 2007; Hiraga et al., 2009) would predict even greater decrements in steadiness for older adults when descending drive from the motor cortex increased during the 30% MVC task and as the fatiguing contraction progressed. Interestingly, error rates in mental math (executive function task) during the fatiguing contraction did not differ across the age groups for the low-cognitive demand sessions and high-cognitive demand, indicating that mental math performance was not diminished in the older adults compared with the young. Although the variability in the CV of torque across the sessions was greater for the older adults than the young, the mean values in CV of torque were similar across the three sessions during the 30% MVC task and increased at similar rates to the young adults during the fatiguing contraction. Time to failure was also similar across sessions for each age group, and the increase in EMG activity, EMG bursting activity, and perceived effort during the fatiguing contraction progressed at similar rates across sessions. Increases in EMG and RPE during a fatiguing contraction are the result of increased descending drive to recruit more motor units in an effort to maintain the required force as the working muscle becomes progressively fatigued (Riley et al., 2008). Thus, while some older adults are clearly more affected than others by the increased cognitive demand during the sustained contractions (Figure 5B), capacity limitations in cortical regions of older adults cannot alone explain the loss of steadiness, especially at the very low forces when descending drive was not large.

Another explanation for the reduced steadiness in older adults as cognitive demand increased is that descending and afferent inputs to the motoneurone pool differed for the older and young adults. One input that likely differed between the young and older adults was monoaminergic drive (Christou et al., 2004). Increased monoaminergic drive to the spinal cord from the brainstem enables motoneurone activation and is essential for exercise (Heckman, 2003), but monoaminergic drive is attenuated in older adults (Meltzer et al., 1998; Reynolds and Meltzer, 1999; Seals and Esler, 2000), potentially leading to decreased motor output and altered responses to increased anxiety and stress compared with young.

### Sex differences in steadiness

Both young and old women demonstrated heightened levels of stress and anxiety, and greater torque fluctuations during the very low-intensity and fatiguing contractions for all three sessions compared with men, regardless of the magnitude of cognitive demand. Similar sex differences in stress and anxiety (Christou et al., 2004) and in torque fluctuations have been shown previously in the upper limb (Yoon et al., 2009; Brown et al., 2010; Keller-Ross et al., 2014a); however, this is the first study to demonstrate increased torque fluctuations in women when performing submaximal contractions of the lower limb with varying levels of cognitive demand. Greater torque fluctuations in women when performing upper extremity tasks have been attributed to strength difference between men and women (Brown et al., 2010), although the mechanism is not known. When exposed to a stressful noxious stimulus prior to task performance, increased torque fluctuations have been attributed to greater activation of central neural mechanisms in response to increased stress and anxiety (Christou et al., 2004); however, in the current study, women demonstrated greater torque fluctuations and reported higher stress and anxiety than men regardless of the magnitude of cognitive demand (i.e., in both low- and high-cognitive demand tasks compared with control). Because both aging and decreased levels of estrogen in postmenopausal women possibly contribute to changes in monoaminergic drive (Meltzer et al., 1998), women may be even more vulnerable than men to diminished motor output with aging (see Figure 5B); however, it remains unclear if greater torque fluctuations in women are related to activation of alternate neural pathways, or a strength-related mechanism.

### Increased variability in fatigability with cognitive demand and aging

Fatigability (time to task failure) of the ankle dorsiflexor muscles was similar across age groups and sessions. Hence, there was no systematic decrease in fatigability when cognitive demand was imposed during the sustained contraction with the ankle dorsiflexor muscles. In contrast, the elbow flexor muscles were more fatigable when high-cognitive demand was imposed in young men and women (Yoon et al., 2009; Keller-Ross et al., 2014a). Similarly, handgrip muscles were more fatigable in both young men and women when high-cognitive demand was imposed for high intensity contractions (Bray et al., 2008, 2012) but not for relatively strong men during a low-intensity sustained contraction (Keller-Ross et al., 2014b). The largest increases in fatigability were related to the initial muscle strength such that weaker participants experienced the greatest increases in fatigability (Yoon et al., 2009; Keller-Ross et al., 2014a). Perfusion associated changes within the muscle in response to a mental-math task (which was used to induce high-cognitive demand) was implicated but only partially explains these findings (Yoon et al., 2009; Keller-Ross et al., 2014a). In contrast to the elbow flexor muscles (Hunter et al., 2004), the ankle dorsiflexor muscles exhibit lesser differences between sub-populations including men and women (Avin et al., 2010), and young and older adults (Kent-Braun et al., 2002; Griffith et al., 2010) and we show here the fatigability of this muscle group is also less responsive to cognitive demand. Christie and Kamen (2009) attribute the lack of difference in fatigability of the dorsiflexor muscles between young and older adults to a lack of difference in motor unit discharge rates, suggesting young and older adults adopted similar neural adaptations during the fatiguing contractions. We found that the increase in EMG activity of the tibialis anterior muscle during the fatiguing contraction was similar across sessions for young and older adults, although older adults had greater EMG relative to the young. Another possible explanation for the different responses in fatigability with and without cognitive demand is a decreased number of corticospinal connections and larger motor unit ratio (motoneurone to fibers) in large lower limb muscles compared with the upper limb (Feinstein et al., 1955). A reduced number of corticomotor inputs to the dorsiflexor muscles relative to the upper limb muscles may minimize the modulating inputs from higher centers imposed with high-cognitive demand and lessen the responsiveness during the sustained fatiguing contractions at the moderate intensity.

While there was no systematic reduction in time to failure of the ankle dorsiflexor muscles as we have observed with the upper limb, older adults, particularly older women, demonstrated significantly more variability in their time to task failure between sessions than young adults (Figure 4). Variability between trials of a motor task can be exacerbated with increased cognitive demand in young people (Lorist et al., 2002) but is often greater with age as shown here. The greater variability in performance with advanced age can be due greater variability in cortical and motor nerve activation during motor tasks (Hunter et al., 2008; Yoon et al., 2008). This greater age-related variability in a motor task when a cognitive task was imposed further demonstrates the important role of cognitive control in determining reliability of performance of motor tasks especially during work-related tasks performed by an aging workforce.

## Conclusion

This study demonstrated that older adults exhibit more variability than young adults in fatigability and less steadiness while performing low-force and moderate isometric with the ankle dorsiflexor muscles. For very low-force contractions, steadiness decreased further as greater cognitive demand increased. The reduced steadiness in older adults compared with the young, may be related to modulation of synergist and antagonist muscles and an altered neural strategy with age. Older adults also exhibited greater variability in steadiness between sessions and in fatigability as cognitive demand was imposed. Increased variability in lower extremity tasks may negatively impact activities of daily living and work tasks that require high-cognitive demand in an aging population. These data also expose differences within an older adult but also between older adults. Our results provide evidence that increased involvement of non-motor cortical areas can disrupt motor performance of low-to-moderate intensity isometric contractions of the lower limb more so in older adults than young adults. These findings have significant implications related to successful aging and performance of activities of daily living with advanced age especially those activities that require simultaneous execution of a cognitive task that involves working memory and maintenance of a static motor task.

## Conflict of Interest Statement

The authors declare that the research was conducted in the absence of any commercial or financial relationships that could be construed as a potential conflict of interest.
